# Circulation of Respiratory Syncytial Virus (RSV) in Poland Between Seasons of 2009/2010 and 2022/2023 Based on SENTINEL System

**DOI:** 10.3390/microorganisms13010140

**Published:** 2025-01-11

**Authors:** Karol Szymański, Anna Poznańska, Katarzyna Kondratiuk, Ewelina Hallmann, Katarzyna Łuniewska, Aleksander Masny, Lidia B. Brydak

**Affiliations:** 1 Department of Virology, National Institute of Public Health NIH—National Research Institute, 00-791 Warsaw, Poland; kszymanski@pzh.gov.pl (K.S.); kkondratiuk@pzh.gov.pl (K.K.); ehallmann@pzh.gov.pl (E.H.); kluniewska@pzh.gov.pl (K.Ł.); lbrydak@pzh.gov.pl (L.B.B.); 2 Department of Population Health Monitoring and Analysis, National Institute of Public Health NIH—National Research Institute, 00-791 Warsaw, Poland; paula@pzh.gov.pl

**Keywords:** epidemic, season, respiratory tract, virus, respiratory syncytial virus

## Abstract

Respiratory Syncytial Virus (RSV) is a prevalent pathogen of the respiratory tract, posing a significant threat to individuals with compromised immune systems, particularly the elderly and neonates in hospital settings. The primary objective of this study was to identify a specific period within the epidemic season during which healthcare providers can anticipate an increased incidence of RSV infections and characterize the epidemic season in Poland. Molecular biology techniques were employed to diagnose samples at Sanitary Stations and the National Institute of Public Health (NIC) in Warsaw. Epidemiological data were collected using the SENTINEL surveillance system. In the 2020/2021 season, there were no reported cases of RSV due to the prioritization of SARS-CoV-2 diagnostics. Before the pandemic, the period of heightened RSV infection risk typically commenced in the 51st week or later, with a statistically significant correlation indicating that a later start was associated with a shorter season duration (*p* = 0.034). In post-pandemic seasons, the temporal distribution of RSV cases exhibited a notable shift, with earlier season onset, peak, and conclusion. Data indicate that RSV is predominantly diagnosed in pediatric populations; however, since the 2017/2018 season, there has been an increase in RSV diagnoses among other age groups. Given the observed shifts in the seasonal peak following the SARS-CoV-2 pandemic, ongoing surveillance is required to ascertain whether these changes are permanent or transient.

## 1. Introduction

Human Respiratory Syncytial Virus (RSV) is a worldwide occurring lower respiratory tract pathogen, causing infections in all age groups. It belongs to the family Pneumoviridae of the Orthopneumovirus genus. In older people, RSV is responsible for a high number of hospitalizations and increased mortality, approaching the values recorded for influenza virus infection. RSV poses a nosocomial risk to infants and people with weakened immune systems. Increased mortality has been observed in patients with bone marrow and lung transplants infected with RSV [1]. RSV infections are one of the leading causes of hospitalization and mortality due to acute respiratory infections (ARIs) in newborns. It is worth noting that more RSV-related deaths are reported in infants aged 1 to 6 months than in newborns and in low- and middle-income countries [2,3,4].

RSV is transmitted through the air, mainly through large respiratory droplets. It can enter the body through the respiratory tract, eyes, and mouth after contact with infected surfaces. RSV can survive for 30 min on the skin surface, for about two hours on fabrics, five hours on gloves, up to seven hours on furniture, and up to twelve hours on non-porous surfaces [5,6,7].

Early symptoms of RSV infection include nasal congestion and discharge. Other common symptoms are cough and, less frequently, fever, which usually have lower grades than in the case of influenza virus infections. In healthy people, symptoms may be mild and affect the upper respiratory tract. In newborns, the elderly, or people with immune disorders, RSV can cause severe infection of the lower respiratory tract [5,6,7,8].

Severe infection can also occur in people with congenital heart defects, cystic fibrosis, and chronic diseases, as well as in people after allogeneic bone marrow transplantation, in infants younger than 6 months of age, and in premature newborns. Diseases of the nervous and muscular systems can also lead to severe infections in elderly people and children [9,10,11].

The rapid diagnosis of RSV infection is crucial to prevent its spread, especially in pediatric hospital wards. The early diagnosis of infection can lead to shorter hospitalization and a reduction in the subsequent use of antibiotics in the case of accompanying bacterial infections [12,13,14]. In the case of diagnostics, studies showed that the most appropriate materials are tracheal secretions and nasopharyngeal aspirates, in contrast to the more widely used nasal swabs [15,16,17].

In the diagnosis of infection, the reference method is RT-PCR, mainly due to its sensitivity, specificity, and ability to distinguish between the A and B variants. Other recommended methods include cell culture, immunofluorescence, and detection of RSV-specific antibodies [18]. The following recommendations are proposed: an annual check of the laboratory’s ability to detect RSV using the EQA panel, use of the RT-qPCR, preseason inspection, and primer matching against current circulating virus strains [19].

Respiratory Syncytial Virus infections usually occur during winter months. In different regions, the start and peak of the season may differ. In temperate regions like Europe, the peak occurs between October and February. On the contrary, in South America, it may be recorded from the 14th week to the 32nd week [20].

In the 2004/2005 season in Poland, the SENTINEL system was introduced by the director of Polish NIC—Professor Lidia B. Brydak [21]. It is a national surveillance system designed to monitor respiratory infections in the general population. Collected data are stored in digital form from the 2009/2010 season onwards. In this paper, data starting from 2009/2010 were analyzed. From the 2014/2015 season, the data were divided into seven age groups for better clarity [22].

The purpose of this study was to present the circulation patterns of Respiratory Syncytial Virus (RSV) in Poland using data from the SENTINEL surveillance system, with a focus on the impact of two distinct pandemics: the A/H1N1/pdm09 pandemic in 2009 and the SARS-CoV-2 pandemic from 2020 onwards. Additionally, this study aimed to identify specific periods within the epidemic season during which healthcare providers can anticipate an increased incidence of RSV infections.

## 2. Materials and Methods

### 2.1. Patient Material and Surveillance System

The test material consisted of nasopharyngeal swabs and bronchial lavage collected from patients diagnosed in Sanitary Stations and in the laboratory of the National Influenza Centre at the National Institute of Public Health NIH National Research Institute.

The results of the molecular tests were reported to the online SENTINEL system by Voivodship Sanitary Epidemiological Stations (VSES)—local divisions of the State Sanitary Inspectorate (Poland is administratively divided into sixteen provinces—voivodships).

The tests included patients in SENTINEL-supervised clinics and hospitals. The number of participating physicians throughout the year varies to around 510 professionals in clinics and hospitals. Information is transferred from physicians through the District Sanitary and Epidemiological Stations to the Provincial Stations. These data are available for further analysis by the National Influenza Centre. Age data were divided into seven groups: 0–4, 5–9, 10–14, 15–25, 26–44, 45–64, and 65+.

### 2.2. Retrospective Data

Retrospective data for RSV infection were obtained from the SENTINEL surveillance system. Data gathered in this system were analyzed at NIC in NIPH NIH NRI to determine seasonal patterns for RSV infections.

### 2.3. Samples Testing Methodology

Each of the 16 provincial stations used kits of their choice to detect respiratory virus infections in diagnosed patients. Tests were performed as routine diagnostics of patients, and all used kits had IVD certificates. For example, stations used FTD Respiratory Pathogens 21—Fast Track Diagnostics (Fast Track Diagnostics Luxembourg S.à.r.l.), Xpert Xpress Flu-RSV (Cepheid, Sunnyville, CA, USA), Vitassay qPCR Flu + RSV + SARS-CoV-2 (Vitassay Healthcare, S.L.U. Spain) tests. There is no reference laboratory for RSV in Poland, so the National Influenza Centre re-tested positive samples sent from VSES. Even though the main focus was influenza, part of the RSV samples was tested as well, confirming that the genetic material of RSV was present in the received samples.

At the NIPH-NIH NRI, Seegene kits (RV12 ACE detection and RV15 one-step ACE detection) were used to detect Respiratory Syncytial virus infection during the period covered in this study. These kits can simultaneously detect infections with 12 or 15 respiratory viruses. The Quiagen Viral Mini Kit and the Promega Maxwell 16 automatic nucleic acid isolation system were used to isolate the genetic material according to the manufacturer’s instructions. The results were interpreted on agarose gel, using thermal cycler software, or by the test manufacturer.

### 2.4. Statistical Analysis

The data were compared using the Kolmogorov–Smirnov test for two empirical distributions (two-sample Kolmogorov–Smirnov test). Due to multiple comparisons, the significance level of 0.05 assumed in this study was adjusted by the Sidak correction.

For 2009/2010–2021/2022, the distribution of the number of cases reported according to the week of the season and the patient’s age was presented using descriptive statistical methods. Cumulative (over time) distribution functions were also analyzed.

For this study, it was assumed that the period of increased risk of infection period (IRIP) starts in the week corresponding to the first decile of the distribution (i.e., the week in which 10% of the season’s cases are reached) and ends in the ninth decile (when 90% of the season’s cases are reached). It was then checked whether the length of the IRIP is related to the timing of the epidemic, i.e., whether it differs systematically between early and late epidemics. The relationship between the starting point of IRIP and its length was analyzed using linear regression. Regression coefficients with standard errors and a determination coefficient were calculated.

### 2.5. Limitations of the Study

This study was limited by a small number of reported RSV samples in the SENTINEL system and no reported data in the 2020/2021 epidemic season. RSV testing was not carried out at the time due to the prioritization of SARS-CoV-2 diagnostics.

We analyzed two seasons after the SARS-CoV-2 pandemic. Although there are publications regarding a slow return to pre-pandemic patterns of RSV seasonality, we do not yet have Polish data from more post-pandemic seasons.

This study was conducted from the perspective of the entire population, considered seven age groups, and did not analyze characteristics of more specific subgroups, e.g., newborns, infants, or seniors.

## 3. Results

**Figure 1:**
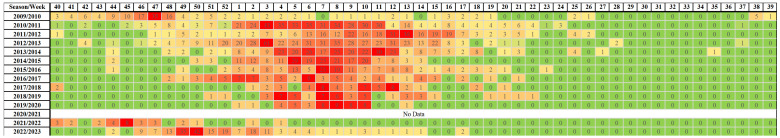
Heatmap with weeks in which positive tests for the RSV were observed. Green means no samples, red means highest numbers of samples.

In this study, fourteen consecutive epidemic seasons were covered. Starting from the 2009/2010 season, when the A/H1N1/pdm09 pandemic occurred, to the 2022/2023 season, after the SARS-CoV-2 pandemic. In total, the report included 2452 positive samples and 18,001 RSV tested samples through this time. The rate of positive samples varied from 4.8% in the 2018/2019 season to 31.7% in the 2009/2010 season. The heatmap in Figure 1 shows the number of laboratory-confirmed RSV virus infections summarized each week.

Each season was covered separately and then compared to others. Seasons with the highest number of recorded RSV infections in the SENTINEL system were 2012/2013 (n = 496) and 2010/2011 (n = 447), then seasons 2013/2014 (n = 278), 2011/2012 (n = 277), 2014/2015 (n = 202), 2022/2023 (n = 201), 2009/2010 (n = 152), and 2015/2016 (n = 143). The lowest number of infections, under 100 in season, was reported in the following seasons: 2016/2017 (n = 79), 2017/2018 (n = 53), 2018/2019 (n = 51), 2019/2020 (n = 48), 2021/2022 (n = 30), and 2020/2021, when no records of RSV were reported to SENTINEL system.

A clear cluster of infections can be observed in a similar period in each of the seasons examined. There is also a clear shift in the peak of infection in the 2009/2010, 2021/2022, and 2022/2023 seasons. They differ from all other epidemic periods and will thus be referred to here as Group One and the remaining seasons as Group Two. In 2009/2010, the highest number of positive samples occurred in the 47th week (n = 35). In the seasons of 2021/2022 and 2022/2023, the highest numbers of positive samples occurred in the 45th (n = 8) and 50th (n = 42) weeks, respectively. The highest numbers of positive samples in the seasons of 2010/2011–2019/2020 clearly occur in a close timeframe each year. The 2021/2022 season shows a lack of consistency in reporting, as evidenced by weeks without confirmed infections (weeks 42, 48, 51, 52/2021 and 1, 3/2022); then, reporting stopped showing positive samples. It is the first season after the reporting gap during the pandemic, which may be responsible for the low number of reports. The 2022/2023 season shows greater consistency in reporting.

Table 1 shows and compares weeks of different stages in the epidemic season. It presents the numbers of the weeks corresponding to the 10th, 50th, and 90th percentiles of confirmed infections for all analyzed seasons. Supplementary numbers of RSV-tested and positive samples were added.

It can be observed (Table 1) that in epidemic seasons of Group Two, 80% of all infections in a given season occurred between week 51 of the year (in the season of 2012/2013) and week 15 of the following calendar year (in the season of 2011/2012). This means that until the 2019/2020 season, the IRIP of the RSV infection generally lasted about 16 weeks in Poland. After the SARS-CoV-2 pandemic, this period shifted and is now observed between week 40 of the year and week 4 of the next second calendar year.

It can also be observed that, in Group Two, half of the registered infections usually occur between weeks 5 (2012/2013, 2016/2017) and 11 (2011/2012) (Table 1). It means that after this period, a similar number of infections should be expected to occur as before, i.e., until weeks 5 and 11, but with a decreasing tendency over time in the subsequent weeks of the epidemic season. In Group One of the epidemic seasons, this time falls between weeks 45 (2021/2022) and 50 (2022/2023) of the calendar year.

Table 1 also shows how long the increased risk of RSV infection lasts in each epidemic season. The most extended period occurs in the 2009/2010 season and the shortest in 2019/2020. In the seasons from Group Two, this period usually lasts 10 to 14 weeks.

**Figure 2:**
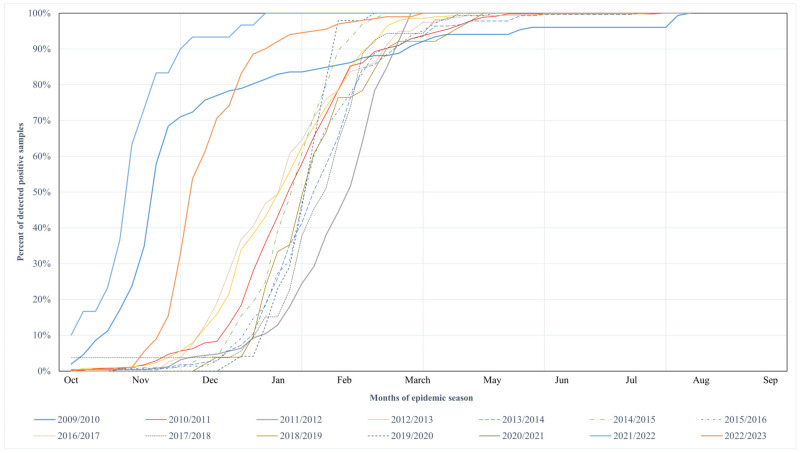
Cumulative distributions of confirmed RSV infection cases in Poland’s discussed epidemic seasons.

The cumulative data for the epidemic seasons are summarized in Figure 2. Data showed the percentage of positive samples for each epidemic season. For the purpose of this paper, the epidemic season was considered to last from October 1st to September 30th of the following year. The 2020/2021 season was a visible exception due to the lack of reported RSV virus infections due to the SARS-CoV-2 pandemic.

Figure 2 is marked in three points: 10%, 50%, and 90% of positive samples. It allows us to observe the dynamics of epidemic seasons. Our analyses focused on data between the 10% and 90% mark, where 80% of all infections were recorded. The first and last 10% were cases recorded before and after peak and were not signs of an epidemic. For this paper, the time when 80% of all infections were recorded was addressed as a period of increased risk of infection. Figure 2 shows that the IRIP in the seasons of 2010/2011–2019/2020 (Group Two) usually starts in December and lasts until February. For seasons 2009/2010 and 2021/2022–2022/2023 (Group One), it starts in October and lasts until December (February for 2009/2010).

As presented in Figure 2, particular cumulative distribution functions for the period 2009/2010–2021/2022 differ markedly. The curves for the seasons 2009/2010, 2020/2021, and 2021/2022 started significantly earlier than in the other seasons. The results of the statistical analysis confirmed this observation. These three distributions are the only ones that differ statistically significantly from any others (*p* < 0.001 after Sidak correction). They were excluded from the relationship analysis between the length of IRIP and epidemic timing.

**Figure 3:**
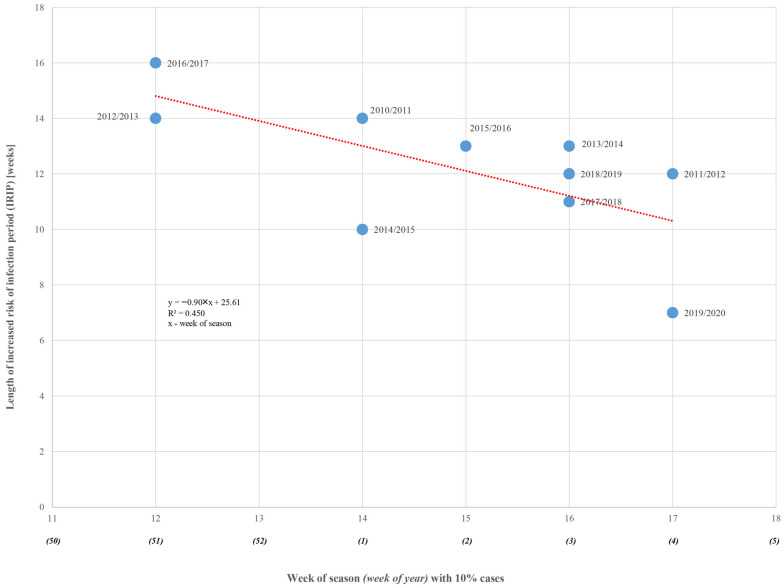
Linear regression of length of IRIP according to week of season onset (red line) for epidemic seasons of Group Two (blue dots).

The length of period with increased risk of infection exhibits a statistically significant linear decreasing relationship with the starting point of IRIP, as shown in Figure 3.

An additional week of delay means the reduction in the IRIP length by 0.90 weeks (*p* = 0.034). The 45% variation in the length of IRIP during an epidemic can be explained by its timing (starting point). This factor cannot be neglected when identifying the expected period of increased risk of infection.

**Figure 4:**
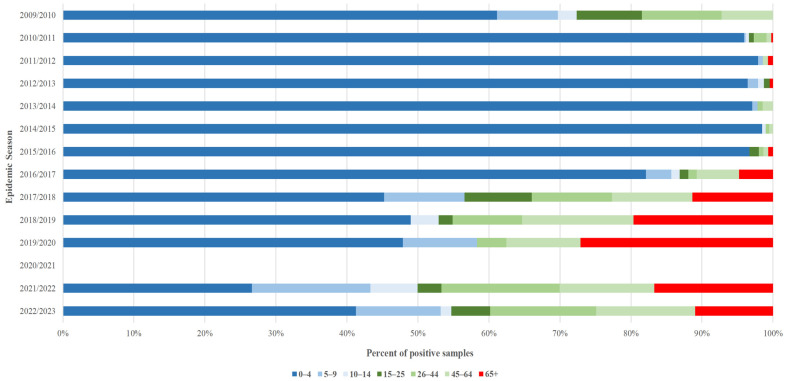
Positive tests for RSV infection divided by age groups in the discussed epidemic seasons in Poland.

Figure 4 illustrates the distribution of cases in the discussed seasons by seven age groups: 0–4, 5–9, 10–14, 15–25, 26–44, 45–64, and 65+.

It can be observed that infections in the 0–4 age group predominate among all age groups and, up to the 2016/2017 season, account for over 50% of all registered infections. An increase in the number of cases reported in the other age groups was visible since the 2016/2017 season, and in 2017/2018, detections in children up to 4 years of age dropped below 50% of all infections. However, the domination of infections in the population under 14 years of age is still observed. An increase in reported infections in people over 65 years of age has been observed since the 2016/2017 season; the highest share in the annual total number of cases (27%) was observed in the 2019/2020 season. The frequency of RSV detections in age groups 45–64, 26–44, and 15–25 also increased since 2016/2017 and notably in the 2017/2018 seasons, varying in different levels for the following seasons.

In the 2020/2021 season, no infections were reported in any age group. Data for this period are not available.

## 4. Discussion

Our main findings are as follows: In Poland, we noticed a divide in epidemic seasons into two groups. Seasons disrupted by the pandemic (2009/2010, 2021/2022, 2022/2023) are marked by a shift in the peak of RSV infections. We also noticed that in a typical season pattern, displayed by seasons of Group Two, a delay in the starting point of IRIP leads to shortening this period. A one-week delay is responsible for shortening by 0.9 weeks.

The observed shifts in the peak of infections and the period of increased risk of infection in the 2009/2010, 2021/2022, and 2022/2023 seasons may be the result of the pandemics of other respiratory viruses at that time. In the 2009/2010 season, a pandemic of influenza A virus broke out, and since then, the subtype has been called A/H1N1/pdm09. The virus displaced the seasonal circulating virus of the A/H1 subtype. The 2021/2022 and 2022/2023 seasons fall immediately after the SARS-CoV-2 pandemic. During the pandemic, VSES laboratories focused on coronavirus diagnostics. Thus, this leads to no reports of RSV infections at that time. This also concerns the influenza virus, both in Poland and worldwide, with a dramatic drop in the number of reported cases, as seen on GISRS charts [23].

The impact of the SARS-CoV-2 pandemic on RSV epidemics is evident worldwide. Other studies reported similar findings to ours regarding the shift in the peak of RSV infections. For instance, a community hospital in Queens County, New York City, USA, observed a shift in the seasonality of RSV in children from mid-October to February to September to January during the SARS-CoV-2 pandemic seasons [24]. Additionally, a study from an urban academic pediatric hospital system in the southern United States documented a shift in RSV seasonality, with off-cycle peaks occurring in the summers of 2021 and 2022 compared to the typical winter RSV season [25]. An analysis of surveillance data from European countries also revealed a noticeable shift in seasonality, with increased RSV activity during inter-seasonal periods, confirming disruptions in the typical patterns associated with this pathogen [26]. There are also publications regarding the shift in the peak of hospitalization in children caused by RSV infection during post-pandemic seasons [27]. Data discussed in this paper come from another source and, to some extent, confirm our findings from the SENTINEL system. Such a change in RSV season timing was also reported by the CDC in the USA [28]. After the pandemic, the first RSV cases were reported at the beginning of the 2021/2022 season. The small number of reports from provincial hospitals and epidemic centers is also evidenced by the lack of consistency in the registration of infections in the winter season, with whole weeks observed without any reported cases. The 2022/2023 season is characterized by greater reporting consistency, which may indicate an increasing number of laboratories reporting infections in the SENTINEL system. This season also sees a slow and steady decline in the number of cases until week 14.

In order to supplement the missing data in the 2020/2021 and 2021/2022 seasons, we analyzed the information provided by the Ministry of Health of the Republic of Poland. The data concern children hospitalized due to RSV infection according to the ICD10 classification: J12.1, J20.5, J21.0, and B97.4 [29]. In the 2020/2021 season, when no information on positive RSV samples was reported to the National Influenza Centre, the Ministry of Health data indicate approximately 1886 hospitalizations due to RSV infection nationwide. In the 2021/2022 season, there were already 15,487 hospitalizations. This again indicates a reporting gap and reorientation of laboratory testing to the SARS-CoV-2 virus, resulting in the lack of reports in the SENTINEL system and a significant decrease in the number of hospitalizations caused by RSV. The more than eight-fold difference in the number of hospitalizations between the two seasons clearly indicates the existence of a factor disrupting the process of reporting, testing, and verifying infections. However, it is also necessary to take into account the many fears of caregivers about the hospitalization of sick children during the COVID-19 pandemic due to the risk of nosocomial infection, lack of contact, etc. An actual reduction in severe RSV infections during this period has also been reported [30]; however, its possible causes (non-pharmacological interventions to reduce SARS-CoV-2 infections or viral interference) are still under debate. Some studies have shown a significant reduction in RSV-associated ALRI hospitalizations during the first year of the COVID-19 pandemic, with a rebound to pre-pandemic levels in high-income countries by 2022. The persistent lower rates in middle-income countries suggest ongoing impacts on healthcare systems and access [31]. There are still no Polish data for this period for adults and people over 65 years old.

The collected data reflect the current knowledge of RSV infections. The virus primarily attacks children and the elderly. This is evident in Figure 4, where the highest infection rates in the seasons covered by this study are observed for the age groups of 0–4, 5–9, 65+ and 45–64. The observed increase in reporting RSV infections in individuals aged 65 years or older may be explained by changes and developments in computerization in reporting respiratory infections in Poland. Since 2016/2017, RSV infections have gotten more focus and attention in VSES and other laboratories that report respiratory tract infections further [32].

In Poland, the SENTINEL system enables the surveillance of the circulating influenza and RSV viruses. General practitioners who joined the system reported infections detected in different provinces of Poland to Sanitary Stations. Then, the National Influenza Centre reports RSV data, such as the number of patients tested for RSV and positive cases, to the TESSy system. These data are also used by both ECDC and WHO.

Establishing a surveillance system for RSV is currently underway and supervised by the WHO [33]. Additionally, ECDC and the WHO European Office launched the European Respiratory Virus Surveillance Summary website (ERVISS). The portal meets the assumptions proposed in the document “Operational considerations for respiratory virus surveillance in Europe” [34]. It allows users to view weekly reports on the epidemiological status of influenza, SARS-CoV-2, and RSV.

A uniform RSV surveillance system would ensure continuous access to relevant resources for researchers and physicians. Transparent access to data, such as that available on the WHO website [35], would provide insight into the current epidemiological situation regarding this virus.

Understanding the epidemiology of RSV is crucial in vaccine development and determining vaccine strategy for those at risk in the future. Currently, two RSV vaccines have been approved by the EU: Arexvy for adults over 60 years old [36] and Abrysvo for pregnant women and people over 60 years old [37]. Adjusting timely vaccination will be beneficial in protecting individuals at risk. Nirsevimab (brand name Beyfortus and Palivizumab (brand name Synagis) are the two drugs used during RSV treatment in the form of monoclonal antibodies, preventing RSV from entering host cells. While both medications have similar usage, there are some key differences between them. Nirsevimab offers higher and more sustained antibody levels and is administered in a single dose. Palivizumab is used mainly in cases of high-risk and prematurely born infants and requires monthly dosage [38,39,40].

Recent studies show the significant impact of Nirsevimab in RSV treatment, reducing the risk of hospitalizations for RSV-associated bronchiolitis in infants [41]. Studies conducted in France, one of the first countries to introduce Nirsevimab in RSV prophylaxis, show a significant drop in RSV-related pediatric burden in hospitals [42].

## 5. Conclusions

In conclusion, the observed shifts in RSV seasonality highlight the dynamic nature of viral epidemics and the need for adaptable and responsive public health strategies. Currently, it remains uncertain whether this shift is permanent or temporary. Thus far, this disruption has advanced the peak of infections from February to October and November. However, our findings corroborate the overall consensus regarding the seasonal shift in RSV infections post-SARS-CoV-2 pandemic and retrospectively extend this observation to the 2009/2010 season during the A/H1N1/pdm09 pandemic. In both instances, a shift in the peak of infection has been documented in the SENTINEL system.

Ongoing surveillance and research are essential to understand these shifts’ long-term implications and to ensure effective prevention and treatment strategies, mainly for vulnerable individuals, infants, pregnant women, and older people. Accurately determining the timing of the RSV epidemic during the winter season will enable healthcare services to better prepare for an influx of patients with respiratory infections. Awareness regarding RSV circulation in the population through the epidemic season and the effectiveness of monoclonal antibodies are crucial in preventing RSV infection and reducing the burden of RSV-related hospitalizations. The timing of infections with RS, influenza, and SARS-CoV-2 viruses overlaps to some extent during the winter. Differentiating between these respiratory viruses in patients presenting with respiratory infection symptoms is crucial for administering appropriate treatment. This differentiation is also significant in the context of immunization windows before the season’s onset. Changes in the timing of the RSV season may impact the effectiveness of vaccines.

## Figures and Tables

**Table 1:** The number of tested and RSV-positive samples during the weeks corresponding to the 10th, 50th, and 90th percentiles of the figure of RSV infections, and length of increased risk of infection period (IRIP) by the discussed epidemic seasons.

Season (Season Group I/II)	RSV Samples		Number of the Week			Length of Increased Risk of Infection (in Weeks)
	Tested	Positive	10% of Cases	50% of Cases	90% of Cases	
2009/2010 (I)	479	152	43	47	16	26
2010/2011 (II)	1692	447	1	6	14	14
2011/2012 (II)	1613	277	4	11	15	12
2012/2013 (II)	3022	496	51	5	12	14
2013/2014 (II)	1843	278	3	8	15	13
2014/2015 (II)	1237	202	1	6	10	10
2015/2016 (II)	1748	143	2	8	14	13
2016/2017 (II)	656	79	51	5	14	16
2017/2018 (II)	963	53	3	9	13	11
2018/2019 (II)	1013	51	3	7	14	12
2019/2020 (II)	605	48	4	8	10	7
2020/2021 (I)	No cases reported					
2021/2022 (I)	295	30	40	45	49	10
2022/2023 (I)	2835	201	48	50	4	9

## Data Availability

Data are contained within the article.
